# Exploring the influence of trust relationships on motivation in the health sector: a systematic review

**DOI:** 10.1186/s12960-015-0007-5

**Published:** 2015-03-31

**Authors:** Dickson R O Okello, Lucy Gilson

**Affiliations:** 1 Health Policy and Systems Division, School of Public Health and Family Medicine, University of Cape Town, Observatory, 7925 Western Cape South Africa; 2 Health Economics and Systems Analysis Group, Department of Global Health and Development, London School of Hygiene & Tropical Medicine, London, UK

**Keywords:** Motivation, Workplace trust, Trust relationships, Health workers

## Abstract

**Background:**

Dedicated and motivated health workers (HWs) play a major role in delivering efficient and effective health services that improve patients’ experience of health care. Growing interest in HW motivation has led to a global focus on pay for performance strategies, but less attention has been paid to nurturing intrinsic motivation. Workplace trust relationships involve fair treatment and respectful interactions between individuals. Such relationships enable cooperation among HWs and their colleagues, supervisors, managers and patients and may act as a source of intrinsic motivation. This paper presents findings from a qualitative systematic review of empirical studies providing evidence on HW motivation, to consider what these studies suggest about the possible influence of workplace trust relationships over motivation.

**Methods:**

Five electronic databases were searched for articles reporting research findings about HW motivation for various cadres published in the 10-year period 2003 to 2013 and with available full free text in the English language. Data extraction involved consideration of the links between trust relationships and motivation, by identifying how studies directly or indirectly mention and discuss relevant factors.

**Results:**

Twenty-three articles from low- and middle-income countries and eight from high-income countries that met predetermined quality and inclusion criteria were appraised and subjected to thematic synthesis. Workplace trust relationships with colleagues, supervisors and managers, employing organisation and patients directly and indirectly influence HW motivation. Motivational factors identified as linked to trust include respect; recognition, appreciation and rewards; supervision; teamwork; management support; autonomy; communication, feedback and openness; and staff shortages and resource inadequacy.

**Conclusion:**

To the authors’ knowledge, this is the first systematic review on trust and motivation in the health sector. Evidence indicates that workplace trust relationships encourage social interactions and cooperation among HWs, have impact on the intrinsic motivation of HWs and have consequences for retention, performance and quality of care. Human resource management and organisational practices are critical in sustaining workplace trust and HW motivation. Research and assessment of the levels of motivation and factors that encourage workplace trust relationships should include how trust and motivation interact and operate for retention, performance and quality of care.

**Electronic supplementary material:**

The online version of this article (doi:10.1186/s12960-015-0007-5) contains supplementary material, which is available to authorized users.

## Background

Health workers (both clinical and non-clinical) form the backbone of any health system. Their motivation and behaviour can significantly influence health system performance [1]. Of concern, therefore, are the reported low levels of health worker (HW) motivation in low- and middle-income countries (LMICs) [2,3]. Low motivational levels have been associated with poor HW practices [4], as well as failure to retain staff [5] and migration of HWs [6-8].

Motivation can be understood as the desire of individuals to act or behave in certain ways. In organisational settings, it can be defined as a behavioural, affective and cognitive process that influences the willingness of workers to perform their duties in order to achieve personal and organisational goals, influencing the extent and level of their effectiveness at work [9,10]. A broad range of theories and frameworks have been developed and used to understand and research this complex phenomenon [10-15]. Yet, for the LMIC health sector, there is still a relatively limited, if growing, body of empirical work about motivation, its determinants and how they interact with other important workplace phenomena across different settings [10,16].

The existing research indicates that HW motivation is influenced by a range of factors. On the one hand, extrinsic motivation—generated when an action or task is performed to receive external rewards or outcomes—is influenced by factors such as remuneration, incentives, rewards, competition, promotion and recognition from superiors [4,14]. On the other hand, factors that influence intrinsic motivation—generated when actions or tasks are performed for internal fulfilment or enjoyment of the activity itself—include autonomy, competency, social interactions, responsibility, cooperation, self-esteem and a feeling of belonging [14,17].

Policy attention worldwide has tended to target the extrinsic motivation of HWs. In LMICs, strategies such as pay-for-performance or establishing conducive work environments have been promoted [18-22], and in higher income settings, new public management strategies such as performance management, audit and marketization are favoured [23-25]. However, interventions focused on extrinsic motivation alone have been argued to lead to a low trust culture that undermines intrinsic motivation [14,18,24], and intrinsic motivation is important because it is specifically linked to positive health worker behaviours, enjoyment of the work itself, the quality of work performed and retention of health workers in current jobs [4,14,17].

**Figure 1:**
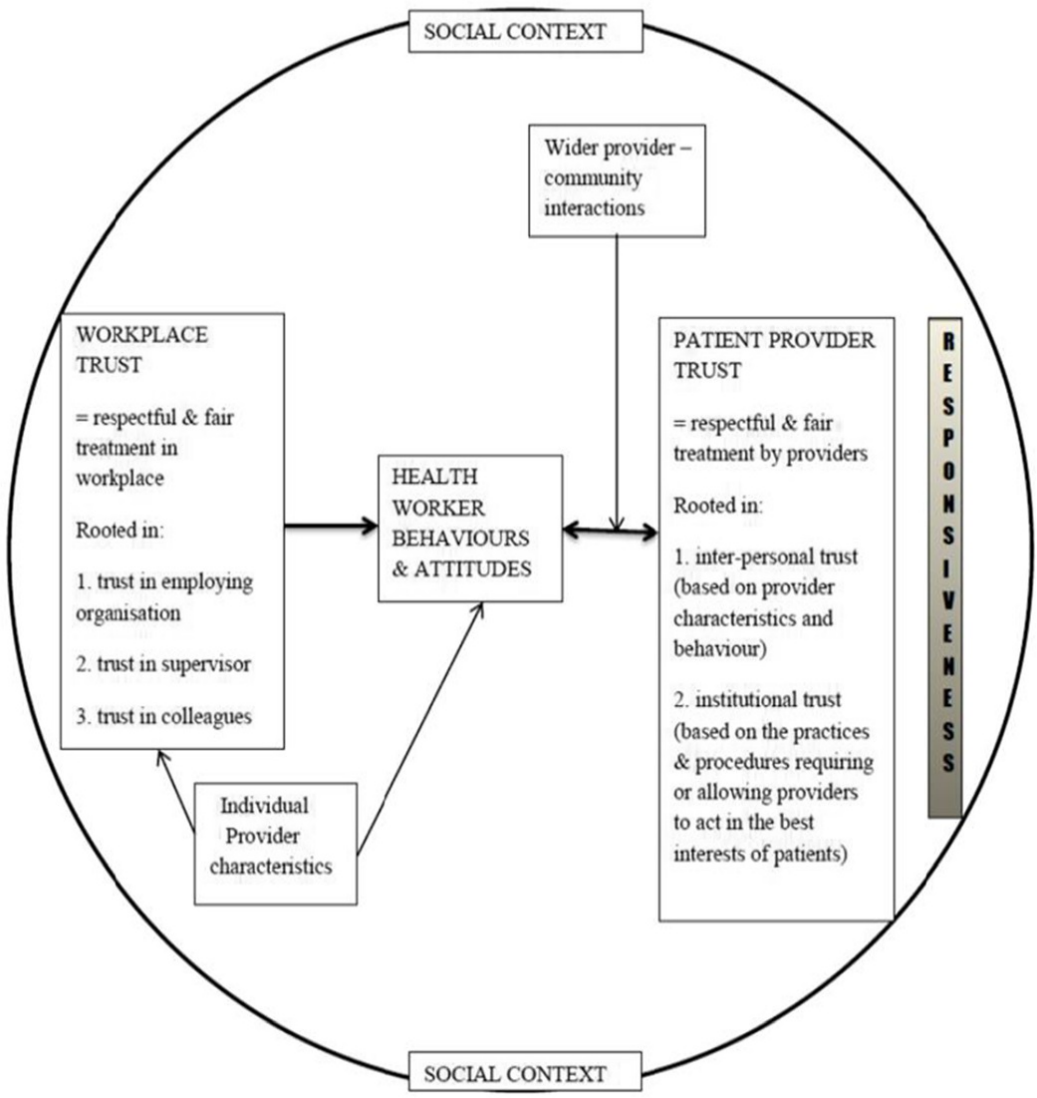
**Trust conceptual framework [** 16 **].**

Therefore, identifying and understanding the intrinsic factors that influence motivation is important for activities aimed at strengthening motivational levels, leading to positive HW behaviour and performance [4]. However, there are few explicit investigations of intrinsic motivation and HW behaviour in the available literature. Possible determinants of intrinsic motivation include social interactions, self-efficacy, competence, autonomy and workers’ sets of values. The organisational literature, in particular, also suggests that trust relationships may have an important influence on intrinsic motivation [26]. Building on this idea, Gilson et al. [16] present a conceptual framework outlining how workplace trust relationships may play out in health care settings (Figure 1).

In broad terms, trust is a relational notion or psychological state that influences individuals’ willingness to act on the basis of the words, motives, intentions, actions and decisions of others under conditions of uncertainty, risk or vulnerability [27-30]. Figure 1 suggests that workplace trust in health care settings is a phenomenon that involves fair treatment and respectful interactions between individuals, and as entailing the provider’s trust in colleagues (linked to teamwork and shared experiences), trust in supervisors (related to personal behaviours and which do have an impact on trust in the organisation) and trust in the employing organisation (influenced by leadership and human resource management (HRM) practices). Such trust relationships enable cooperation among HWs and their colleagues, supervisors, managers and patients and may act as a source of intrinsic motivation. The factors that allow for the development of workplace trust also allow patients to presume that HWs are adequately competent and will adopt the positive attitudes that enable their health care needs and expectations to be met [27-29,31]. Similarly, positive engagements with patients themselves also motivate HWs, leading to the interaction between workplace trust and provider–patient trust [16]. This framework is useful in analysis and identification of interpersonal and organisational elements of the dimensions of trust relationships, including provider–patient trust.

Broader organisational literature suggests that possible influences over these four sets of relationships include communication standards, feedback mechanisms, competence, performance appraisal and reward systems, job security and organisational support and procedures—including decision-making practices [32]. These determinants affect the nature of interpersonal trust relationships and may present values that shape workers’ attitudes and behaviours, thus having an influence on their motivation [27,29,32,33].

Against this background, this review seeks to answer the question: Do workplace trust relationships influence the motivation of HWs, and if so, how? Considering available literature on the determinants of HW motivation, the review examines whether workplace trust is identified as an influencing factor in such studies, and if and how the four trust relationships of Figure 1 are found to influence motivation.

## Methods

Intended primarily to map the available evidence base, the review process followed standard qualitative systematic review steps [34-37]. Formal ethical considerations or confidentiality procedures were not needed for this review because the authors accessed and utilised only publicly available and published data.

### Search strategy

Five electronic databases considered as sources of relevant literature on HW motivation were searched. These are PubMed/MEDLINE, Cumulative Index of Nursing and Allied Health Literature (CINAHL), PsycINFO, Africa-Wide Information and Scopus. CINAHL, PsycINFO and Africa-Wide Information were searched independently via EBSCOhost. The keywords and MeSH terms for the review included ‘Motivation’, ‘Job Satisfaction’, ‘Attitude of Health Personnel’, ‘Retention’, ‘Trust’, ‘Workplace trust’, ‘Relationships’, ‘Interpersonal relations’, ‘Health Personnel’, ‘Health Sector’ and ‘Health Worker’. These terms, in addition to other words, were applied appropriately to each database as outlined in the search algorithm in Additional file 1. The identified studies were then transferred to a reference manager, RefWorks (Copyright© 2009), to save and facilitate scanning of the titles and abstracts for the inclusion and exclusion criteria.

### Article selection

For inclusion, the article had to report findings of empirical research on the determinants of motivation of any cadre of clinical and non-clinical health workers. The electronic search and selection included evidence from LMICs and high-income countries (HICs). All relevant empirical studies that utilised qualitative, quantitative and mixed methods approaches were considered for this review. Original and review journal articles with available free abstract and full text were identified from the databases.

**Figure 2:**
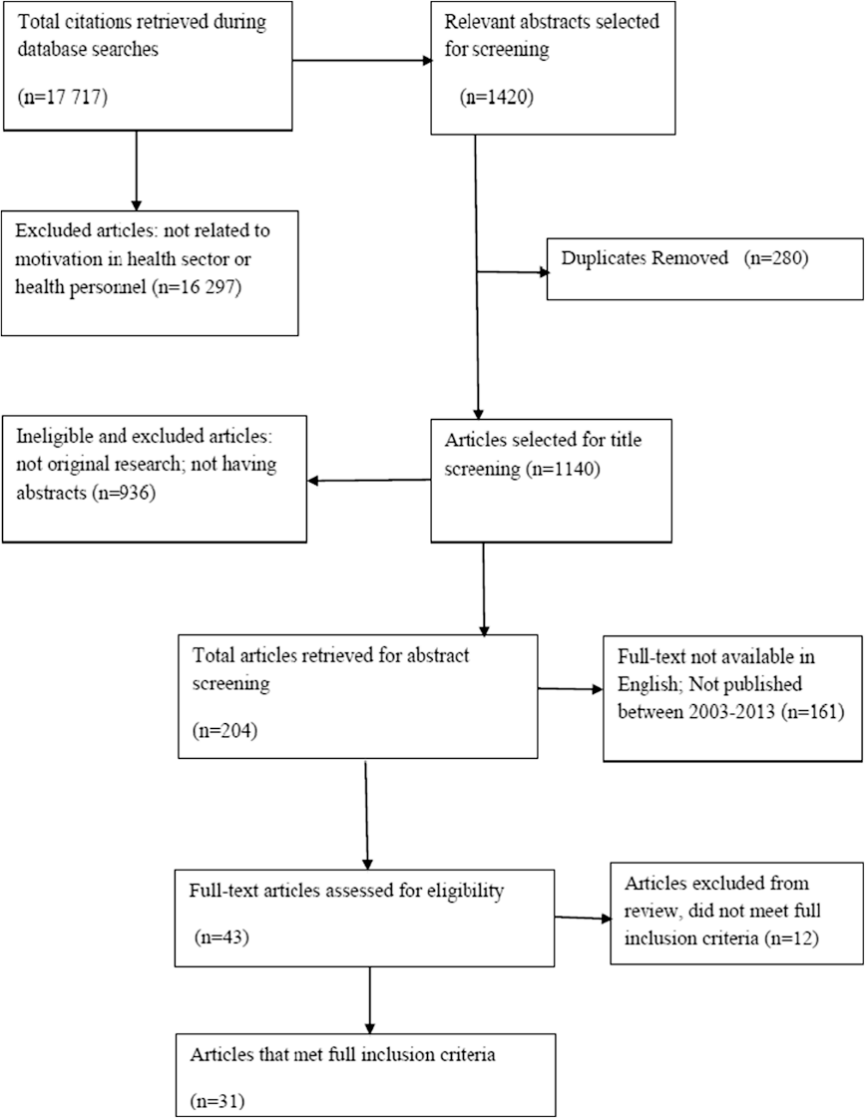
**Search flow chart.**

The inclusion criteria also limited studies to the period from 2003 to 2013, a period deemed appropriate to encompass the most recent relevant literature, and to papers published in English. The exclusion criteria were 1) studies not related to HW motivation and/or motivation in the health sector; 2) studies published prior to the year 2003; 3) studies published in languages other than English; 4) articles or citations without abstract; and 5) studies that did not provide information on HW motivation in the full text. To identify relevant studies for review, the titles and abstracts were screened against the inclusion and exclusion criteria after removing duplicates from the combined search output, followed by full-text reading of identified studies. The search PRISMA or flow chart is presented in Figure 2.

### Quality review and data extraction and analysis

Reviewers acknowledge difficulty in appraisal of qualitative studies and have suggested criteria with specified guidelines for judging suitability of studies for inclusion in qualitative systematic reviews [35,38,39]. The Critical Appraisal Skills Programme (CASP) criteria for assessing study rigour, research methods, credibility and relevance were used to judge the quality of the papers selected for this review [40]. Twelve papers out of the 43 initially selected for review were deemed to be of poor quality against the CASP criteria and were excluded from further review.

The data extraction form (Additional file 2) structured in line with the motivation framework of Franco and her colleagues was used as a data registry and as a guide for identification of the determinants of motivation [10]. The workplace trust framework by Gilson and colleagues was then employed to identify and categorise those determinants linked to workplace trust [16]. This combination allowed for a fairly open data extraction approach, followed by a more focussed description and analysis. The extraction involved line-by-line coding during detailed reading of the findings and discussion sections of each selected paper, to identify factors that determine motivation and issues about how trust relationships influence motivation.

The authors used thematic analysis [36,38,41,42] to identify, map and categorise the data from the selected articles. Line-by-line reading of the papers allowed identification of specific experiences directly or indirectly important in motivation. Consideration was also given to whether study findings directly or indirectly mentioned and discussed factors or experiences that are relevant to trust relationships—for example, management support, job security, job stability, supervision, involvement in decision making, promotion, communication, feedback mechanisms, trust, rewards, respect, recognition, appreciation, transparency, confidence, fairness and other organisational processes and resources [32]. These experiences and words formed the basic codes that allowed extraction of data. The identified experiences were initially categorised by the different sets of possible workplace relationships and then grouped by whether they were identified as positive or negative influences over motivation, as well as by common themes of influence.

The first author was responsible for article searches, identification, synthesis, analysis and the write-up of this article. In supervising the whole review process, the second author specifically supported search strategy formulation, article selection, analysis of the articles and revision of the final draft of this paper.

## Results

### Characteristics of selected articles

More than 17 000 citations were retrieved from the initial search (Additional file 1). Following a screening of the titles and abstracts, 43 articles were selected for full text reading. After full text reading, finally, thirty-one articles that met the full inclusion and quality criteria were considered for this review as indicated in Figure 2. A summary of the included articles is outlined in Table 1. Twelve articles that clearly met one or more of the exclusion criteria and whose relevance and quality was judged as poor based on the appraisal tool were eliminated (see Table 2).

**Table 1:** **Summary of articles under review**

Author(s) and year of publication	Country of study	Study objective(s)	Study population	Methodology	Data analysis	Study findings
Agyepong et al. 2004 [67]	Ghana	Description of factors that influence HW job satisfaction and motivation	HWs across public health facilities in the Greater Accra region	Continuous quality improvement, structured questionnaires	Statistical/Pareto analysis	Workplace obstacles such as salaries and lack of equipment, tools and supplies influence HW motivation.
Alhassan et al. 2013 [56]	Ghana	To explore the quality of care and patient safety situation in health facilities accredited by the Ghanaian National Health Insurance Authority and identify associations with HW motivation	Clinical and non-clinical HWs	Structured questionnaires based on in-depth interviews	Statistical analysis with STATA version 12	Low motivational levels. HWs dissatisfied mainly with non-financial incentives including transport to work, career development prospects and poor relations due to resource inadequacy at the workplace. Membership in professional associations had a positive influence on their professional practice.
Campbell et al. 2011 [72]	Zimbabwe	To examine nurses’ motivation and frustration in the context of the roll-out of antiretroviral treatment in Zimbabwe	Nurses, HIV counsellors, nurse-pharmacist, nurse assistant and administration clerks	In-depth interviews, focus group discussions (FGDs) and ethnographic observation	Thematic analysis	HWs’ motivation to provide high-quality antiretroviral treatment influenced by patients’ emotional improvement and recoveries, patient commitment to treatment and personal experiences for compassion. HWs demotivated by staff shortages, inadequate medicines and equipment, low salaries and losing patients’ confidence.
Chandler et al. 2009 [58]	Tanzania	To evaluate factors that affect motivation and levels of motivation among non-physician clinicians	Non-physician clinicians	Interviews and FGDs, quantitative survey instrument	Thematic analysis and statistical analysis	Salary ranked as the most important source of motivation. Non-financial factors that influence motivation include social status expectations, working environment and relationships with different cadres.
Dickin et al. 2011 [53]	USA	To identify important sources of motivation to facilitate the development of strategies to enhance community HW motivation and enhance performance and program effectiveness	Community nutrition educators	Qualitative in-depth interviews, quantitative surveys and supervisor questionnaire	Coding using the constant comparative approach and thematic analysis using ATLAS.ti software	Community nutrition educators mentioned several factors as motivators, including interest in educating people on food and nutrition, caring relationships developed among participants and the educators, freedom to make job-related decisions, relationships with supervisors and the team, and good health benefits
Dieleman et al. 2003 [50]	North Viet Nam	Perceptions on what motivates and demotivates HWs, perceptions of HWs and managers on HRM tools, perceptions of community members	Policy makers and managers; HWs (ass. doctor, nurses, midwives); community members	Semi-structured exit interviews, FGDs	Qualitative data analysis	Financial and non-financial incentives influence motivation, especially appreciation by managers, colleagues and community, stable job, income and training
Dieleman et al. 2006 [62]	Mali	To describe HW motivation and demotivation factors and match motivators with implementation of performance management	Managers, HWs, village committee members	In-depth interviews and FGDs, cross-sectional descriptive survey (questionnaire)	Statistical analysis (SPSS). Triangulation of qualitative data and survey	Salary, responsibility, training, recognition and rewards. Performance management like job descriptions, supervisions, continuous education and performance appraisal influences motivation
Franco et al. 2004 [51]	Jordan and Georgia	Identify motivational determinants in LMICs’ public health sector. Identify interventions and strategies for health care reforms to facilitate HW motivation	Two public hospitals in each country. Key informants at hospital and Ministry of Health managers, supervisors, HWs and patients	Contextual analysis (qualitative interviews and document reviews). 360-degree assessment (qualitative and quantitative, structured interviews). In-depth analysis	Statistical analysis using psychometric scales	Financial and non-financial determinants at individual, socio-cultural and organisational levels.
Greenspan et al. 2013 [61]	Tanzania	To explore sources of community HWs’ motivation to inform programmes in Tanzania and similar contexts	Community HWs	Semi-structured in-depth interviews	Thematic analysis	Levels of motivation identified as individual, family, community and organisational. Families and communities providing moral and financial support, recognition and encouragement. At the organisational level, monetary support, job security, tools and supplies for work, training and supervision considered as motivational factors.
Hegney et al. 2006 [52]	Australia	To identify intrinsic and extrinsic work values that influence job satisfaction	Public, private and aged care nurses	Survey questionnaire	Statistical analysis (SPSS). Thematic analysis of qualitative data from the questionnaire	Remuneration, rewards, working conditions, work stress, autonomy and social relations at work affect job satisfaction and intention to leave employment.
Kahler et al. 2012 [47]	Denmark	To explore motives for choosing employment at either public or private hospitals in a group of Danish surgeons. To examine effects of organisational characteristics on motivation	Surgeons	Qualitative interviews	Phenomenological/thematic analysis	Motivational factors that were identified include possibility to provide optimal patient care, having influence on the job, challenging work tasks, relationships with colleagues and ideological reasons.
Kok and Muula 2013 [69]	Malawi	To identify factors that influence motivation and job satisfaction of health surveillance assistants in Malawi, in order to inform development of strategies to influence staff motivation for better performance	Health surveillance assistants	Key informant interviews, FGDs and a group discussion with supervisors. Questionnaire for household survey of surrounding community	Coding framework for qualitative data analysis. Statistical analysis using SPSS version 17 for quantitative data analysis	The study found that salaries were low with no opportunity for promotion and that there were heavy workload with no job descriptions and lack of opportunities for training. The workers were further demotivated by lack of transport, lack of recognition from supervisors and management, limited supervision and lack of communication.
Kontodimopoulos et al. 2009 [43]	Greece	To identify motivational factors of health professionals and to determine if the factors differ in the public and private sectors	Doctors, nurses and office workers in 13 hospitals	Quantitative: 28-item questionnaire survey	Statistical analysis (SPSS)	Both monetary and non-monetary factors determine HW motivation. Achievement, remuneration, working relationships with co-workers and job attributes influence HW motivation. Health professionals in private hospitals were motivated more than those in public hospitals.
Kudo et al. 2010 [44]	Japan	To examine associations between work motivation and job satisfaction among Japanese nurses to improve their motivation	Nurses	Self-administered questionnaires	Statistical analysis	Nurses do feel motivated not only by money but also by their work as specialists, workplace safety, relationships with superiors, work–life balance, relationships among themselves and communications with physicians.
Kyaddondo and Whyte 2003 [63]	Uganda	To study the effect of decentralisation and policy reforms on HW motivation	4 health units, health managers, health unit management committee and health unit workers	Interviews, document reviews, FGDs and observation	Qualitative data analysis	Decentralisation is critical to professional autonomy, recognition, coping strategies and demotivation
Lambrou et al. 2010 [45]	Cyprus	To investigate how medical and nursing staff of Nicosia General Hospital is affected by specific motivation factors, and the association between motivation and job satisfaction. To determine the motivational drive of socio-demographic and job-related factors in terms of improving work performance	Doctors, dentists and nurses	Cross-sectional survey (questionnaire)	Statistical analysis	Achievements were ranked as the top main motivator followed by remuneration, co-workers and job attributes. Female HWs were more motivated by remuneration compared to male HWs. Professional relationships with colleagues and supervisors was identified as a source of satisfaction and motivation.
Leshabari et al. 2008 [70]	Tanzania	To measure the extent to which HWs at Muhimbili National Hospital were satisfied with their work. To identify factors associated with low motivation in the workplace	Doctors, nurses, auxiliary clinical workers, and administrative and support staff.	Structured interviews (cross-sectional study)	Statistical analysis (SPSS)	HW dissatisfaction and low motivational levels due to low salaries, lack of equipment and drugs, inadequate performance evaluation and feedback, poor communication channels in different units (and between workers and management), lack of participation in decision making, lack of concern for HWs’ welfare by management.
Malik et al. 2010 [60]	Pakistan	To identify the determinants of job motivation among physicians	Physicians from public primary, public secondary and public and private tertiary health facilities	Open-ended questions, semi-structured self-administered questionnaires and in-depth one-on-one interviews	Thematic analysis and statistical analysis	Motivating factors mainly intrinsic and socio-cultural, including serving people, respect, career growth and personal safety. Demotivators included few opportunities for higher qualifications, resource unavailability, poor supervision and poor interpersonal relations.
Manafa et al. 2009 [64]	Malawi	To explore how clinical health officers are managed and motivated and the impact this has on their performance	District managers, Ministry of Health officials and different cadres of HWs	FGDs, key informant interviews	Thematic analysis	Continuous education, career progression, supervision and feedback on performance considered inadequate by HWs, while performance appraisals and clear job descriptions were non-existent. District managers did not perceive these factors as having an impact on motivation.
Manongi et al. 2006 [66]	Tanzania	To explore the experiences of HWs working in the primary health care (PHC) facilities in Kilimanjaro Region. To identify areas for sustainable improvement to services provided by HWs	Multiple cadres of HWs in PHC facilities in 3 districts, district medical officers	2 FGDs in each of the 3 districts, semi-structured interviews	Thematic analysis	Staff shortages, poor supervision from managers, lack of transparency in career development opportunities.
Mathauer and Imhoff 2006 [57]	Benin and Kenya	To assess the role of non-financial incentives for motivation	Doctors and nurses in rural areas, Ministry of Health officials	Semi-structured qualitative interviews, FGDs	Statistical (SPSS) analysis of quantitative and coded qualitative data	Appreciation of professionalism, recognition, career development, supervision, participation in decision making, performance appraisals and team-based performance management influence motivation.
Mbilinyi et al. 2011 [71]	Tanzania	To explore the challenges generated by HIV care and treatment and their impact on HW motivation in Mbeya Region	Different cadres of HWs	Qualitative in-depth interviews	Qualitative framework analysis and thematic analysis	Demotivation due to risk of contracting HIV and tuberculosis; lack of acknowledgement and appreciation from managers and community; staff, drugs and essential supplies shortages; poor infrastructure; favouritism; and relationships between HWs and colleagues and with the community.
Mbindyo et al. 2009 [59]	Kenya	To explore contextual influences on HW motivation	HWs and key informants in 8 rural district hospitals	Individual in-depth interviews, small group interviews, FGDs and observation	Thematic analysis using NVivo 7 software	Management practices at the hospital level influences HW motivation. Supportive leadership fosters good working relationships and improves motivation through incentives, promotions, performance appraisals and good communication processes. Poor schemes of service demotivate.
Mubyazi et al. 2012 [65]	Tanzania	To describe the supply-related drivers of motivation and performance of HWs in administering preventive treatment of malaria at ante-natal clinic services in public and private facilities	Clinical officers, nursing officers, midwives, laboratory personnel, nurse auxiliaries, public health nurses, maternal and child health aides, and health assistants	Field observations, document reviews, in-depth interviews and questionnaire with a mix of closed- and open-ended questions	Content analysis of qualitative data and statistical analysis of quantitative data (STATA 8.2)	Dissatisfaction and performance constraint due to poor working environment, understaffing, poor supervision, limited career development opportunities and poor health facility infrastructure and staff houses. HWs in private facilities more motivated compared to those in the public facilities.
Newton et al. 2009 [74]	Australia	To identify what motivates individuals to engage in nursing career	Registered nurses and nurse managers	Semi-structured interviews, surveys and fieldwork observation	Thematic analysis	Desire to help, a caring motive, sense of achievement and self-validation were identified as factors that influence nurses’ motivation.
Peters et al. 2010 [46]	India	To identify important aspects of HW satisfaction and motivation in 2 Indian states in both public and private sectors	Doctors, nurses and other HWs	Cross-sectional questionnaire survey using a 17-item instrument	Statistical analysis	Non-financial motivators identified include good working relationships with co-workers, workplace environment and opportunities for personal development, recognition and autonomy. Good financial remuneration also considered as an important motivator.
Prytherch et al. 2012 [55]	Tanzania	To explore HWs’ understanding of motivation. To explore factors that encourage or discourage providers of maternal and newborn health care in rural areas. To explore factors that influence rural HWs’ performance and job satisfaction	Maternal and newborn HWs in rural settings	In-depth interviews	Thematic analysis using NVivo v9 software	HWs had understandings of motivation. Identified motivators or source of satisfaction included community appreciation, perceived government and development partner support and on-the-job learning. Discouragements were related to poor security, health and safety, lack of job descriptions, problematic supervision and performance appraisal.
Prytherch et al. 2013 [49]	Burkina Faso, Ghana and Tanzania		Maternal and neonatal health care providers, policy-level informants, district- and facility-level managers	In-depth interviews	Thematic analysis	Most community HWs mentioned that they were drawn to the profession for altruistic reasons. Other than salaries and incentives, good relationships with managers, supervisors, patients and the community also influenced motivation. Problems in rural areas like availability and cost of water and electricity, difficult working conditions, distance to one’s family and lack of information demoralised the HWs.
Razee et al. 2012 [48]	Papua New Guinea	To investigate social factors that lead to motivation of staff working in and affect performance of lower level health facilities in rural PNG	Health extension officers, community HWs and nursing officers	Face-to-face semi-structured in-depth interviews	Thematic analysis (NVivo 8.0 software)	Good relationships with staff, community and friends, and cooperation and responsibility from the patients were mentioned as motivators. HWs were unhappy with poor communication and interpersonal relations, lack of trust and respect, societal expectations around women, workplace safety and security.
Siril et al. 2011 [54]	Tanzania	Assessing individual and site-related factors associated with HW-reported stress, motivation and perceived ability to meet the needs of patients enrolled in PEPFAR-supported public sector HIV clinics. To identify areas for improvement to promote staff retention of HWs in resource-limited settings.	HWs at HIV care and treatment centres	Self-administered questionnaire	Statistical analysis in SAS 9.1.	Half of the respondents felt motivated to perform their jobs. Motivation was influenced by specialised training, adequate supervision, ability to meet patient needs, teamwork with good understanding, respect and good communication among staff members, good working environment and availability of equipment and supplies. Lack of feedback on performance demotivated HWs.
Zinnen et al. 2012 [68]	Tanzania	To contribute to empirical evidences on human resources for health motivation by assessing the role of financial and non-financial incentives and measuring the reasons to stay working in rural areas	Different cadres of HWs and district/council health management team	In-depth interviews with key informants, structured questionnaires with closed- and open-ended questions, and document and report review	Coding and analysis using MAXQDA software version 2007. Epi Info software version 3.5.3 for quantitative data analysis	High staff stability in public health facilities. HWs motivated by better job security, salary and retirement benefits, supportive supervision and support for career development. Dissatisfaction was due to inadequate work equipment, staff shortages, heavy workload and favouritism in allocations for allowances and further training.

**Table 2:** **Studies excluded from the review**

Study	Reason for exclusion
Agyei-Baffour et al. 2011 [75]	The paper assessed the influence of intrinsic and extrinsic motivation on willingness to accept postings to rural areas among medical students. The study group did not represent HWs and thus did not meet the full inclusion criteria.
de Guzman et al. 2009 [76]	This article was a phenomenological study of motivation and attitudes of six nurses towards geriatric care. The findings and discussion lacked relevance to the review hence exclusion because they failed to meet the quality criteria based on the CASP tool.
Gambino 2010 [77]	The paper reported findings from a study of the relationships between registered nurses’ motivation for entering the profession, occupational commitment and intent to remain. It utilised a mix of students and nurses at a university medical centre hence was excluded because it did not meet the full inclusion criteria. It also failed to meet the quality criteria based on the assessment/quality appraisal tool, CASP, the findings dwelt on transformative change and were adjudged irrelevant to this review.
Helmink et al. 2012 [78]	The article reported findings from a study that examined factors explaining motivation among HWs to implement a single programme to support prevention and treatment of type 2 diabetes mellitus. Despite having a strong theoretical and methodological background, the findings lacked credibility and relevance to the review question and objectives.
Imai et al. 2010 [79]	The article was based on a study on factors associated with motivation and hesitation of health professionals during a public crisis in Japan. It was excluded because it only considered motivation during a crisis and the findings on motivation were not credible and relevant to this review when subjected to the CASP tool.
Kamanzi and Nkosi 2011 [80]	The paper explored factors that influence the motivation levels of nurses working in a university teaching hospital. The data collection and analysis were not clearly outlined hence lacked rigour. The results were listed without any clear explanation and discussion.
Leonard and Masatu 2010 [81]	The study explored intrinsic motivation among HWs for evidence on professionalism. The findings were related to the knowledge of clinicians in relation to how they perform their duties and not motivational factors as per theoretical framework. The findings lacked credibility and were not relevant to this review.
Lopes and Delellis 2013 [82]	The study on understanding the motivations of the multi-generational physician assistant workforce used convenient sampling of conference attendees. The study lacked rigour in research methods.
Minai and Almansour 2013 [83]	The study investigated factors influencing job satisfaction and motivation of nurses in the male nurse-dominated environment which were not clearly explained. The findings were not well presented and discussed and hence lacked credibility and relevance.
Mubyazi and Njunwa 2013 [84]	Despite having a rigorous methodology, the study on perceived impact of health sector reforms on motivation of HWs and quality of care did not investigate the motivational determinants and hence failed to meet the full inclusion criteria.
Negussie 2012 [85]	The study investigated the relationship between rewards and incentives and nurses’ work motivation but was not well presented and meaningful to the study objectives. It also lacked relevance to the review question.
Serneels et al. 2007 [86]	The study aimed to understand the role of intrinsic motivation in influencing HWs’ choice to work in faith-based institutions in rural areas. The study participants were nursing and medical students. Excluded because of not meeting the full inclusion criteria (study participants were not HWs)

The selected articles were studies carried out in Africa (19), Asia (4), Europe (4), Australia (2), United States of America (1) and Oceania (1). Of the African studies, Tanzanian experiences (9) represented a third of the studies reviewed. Three articles were multi-country studies. The reason why Tanzania had a high number of articles on HW motivation could not be deduced.

With regard to research methods, 14 of the selected articles used qualitative approaches, 10 used quantitative approaches while 7 utilised a mix of both qualitative and quantitative approaches. With respect to study participants, half of the articles under review focused on all cadres of HWs in the respective countries of study, with some including informants from ministries of health. Four articles specifically dealt with motivation among nurses, five all cadres of HWs and patients or community members, four community HWs only, one practising surgeons and one non-physician clinicians.

### Major factors related to trust and motivation

The reviewers identified motivational factors that directly or indirectly relate to workplace trust relationships. Overall, it was explicitly noted in 21 of the 31 articles that HW trust relationships with their colleagues, supervisors, managers, employing organisation or patients influenced motivation and/or performance. Important motivational factors that were directly and indirectly linked to the presence and influence of trust relationships include respect; recognition, appreciation and rewards; supervision; teamwork; management and welfare support; professional autonomy and professional association; communication, feedback and openness; and staff shortages, heavy workload and resource unavailability. Although not the focus of this review, consequences of workplace trust over, for example, retention, performance and quality of care were also identified and these findings are later presented in the Discussion section of this paper.

The four trust relationships of Figure 1 were confirmed in this review, that is, trust relationships with colleagues, managers and supervisors, employing organisation and patients. In the following section of the review findings, the key influences over HW motivation, as extracted from the papers, are reported against each of the four workplace trust relationships. Finally, a discussion and conclusions to this review are presented.

### Trust relationships with colleagues

Good working relationships and trust between HWs and their colleagues were explicitly considered as strong motivational factors in seven articles [43-49]. In addition, other articles implied that HWs believed support from colleagues, professionalism, high levels of teamwork with respect, and understandings between colleagues were both evident in their workplaces and motivating [50-54]. A survey among medical and nursing staff in Cyprus found that positive HW relationships with co-workers, evidenced by appreciation and respect between doctors and nurses, enhanced workplace trust and was ranked as the second strongest motivator after remuneration [45].

A qualitative study reporting on the influence of social factors on motivation of HWs in Papua New Guinea revealed, meanwhile, that relations between colleagues was particularly important for motivation: “*If I am happy with the staff, my staff relationship and the community, and also the friends I work with, they are helping, it motivates me to continue to work here …..*” [48] (p. 830). This quote highlights the consequence of workplace trust relations for retention, as HWs believed that co-workers in rural areas provided emotional help and support in times of stress. Studies reported that trusting relations developed through professionalism and ability to consult with colleagues when not sure of procedures or treatment guidelines were motivating [46,47,49,55,56]. This was seen as good for HW performance and quality of care because of the possibility of sharing professional knowledge for the effectiveness of clinical and interpersonal care. For example, a multi-country study quoted a female Burkina Faso auxiliary midwife from an in-depth interview, commenting on good relations between staff: “*I feel comfortable working here….. In most instances I can rely on my experience. But if I am not sure then I do not worry but ask my colleagues for their help*” [49] (p. 7).

Inversely, five papers reported poor trust relationships between colleagues as sources of demotivation. HWs did not trust their colleagues and listed reasons for poor relations as lack of collegial support, disrespect, poor teamwork and being ridiculed when seeking assistance, leading to them not offering quality services and taking out their frustrations on patients [49,57-60]. It was reported that envy among colleagues, an indication of lack of trust and poor relations, was demotivating: “*If one colleague tries to work hard, others gang up against him*” (male clinical officer in Kenya); “*making efforts on your own creates envy and you will face obstacles*” (female nurse in Benin) [57] (p. 13). Suspicions between colleagues were reported to have an undermining effect on workplace trust [49]. Further, workplace trust was undermined by poor interpersonal relationships between different cadres, where clinical officers in Kenya thought that nurses and doctors were against them [59].

### Trust relationships with supervisors and managers

In this review, the authors used the term ‘*supervisors and managers*’ to denote individuals responsible for controlling, administering, directing, overseeing, guiding and assisting HWs in health care settings as outlined in the articles under consideration. Based on the evidence considered, it was difficult to delink supervisors from managers. The manner in which the supervisors carry out their roles also determines the relationships between them and the HWs.

Positive trust relationships with supervisors and managers were clearly associated with HW motivation in eight articles [44-48,53,54,61]. One study from Japan [44] and one from Tanzania [61] specifically found that trusting the supervisor to provide information and instructions, identify areas for improvement, help with problem solving and give additional training was responsible for good relations and motivated workers to effectively perform their duties. Health workers in HICs believed that the ability to work independently was highly motivating because they were likely to earn the supervisor’s trust, as articulated in one of the papers: “*And I think we’ve all developed a trust with her [supervisor] so she knows that we’re going to do a quality kind of thing. She comes and checks what we’re doing from time to time and so she has a general sense that we have the ability to do that sort of thing on our own*” [53] (p. 265). This provided evidence that workers’ degree of control and ability to make informed decisions influenced relationships with their supervisors and played an important role in boosting HWs’ motivation and performance. Indicators of trust relations such as being given greater responsibility, recognition, appreciation and respect by managers and colleagues were linked to good workplace trust relationships, between HWs and their managers and supervisors, that influenced motivations [43,49,50,57,58,60,62].

However, nine studies identified poor supervision as a cause of stressful relationships between HWs and their supervisors within the workplace and as demotivating [55,57,59,63-68]. The relationships were poor when supervisors did not appreciate workers and their actions were geared towards fault-finding [59], sometimes blaming workers without considering the poor working conditions [55]. Studies identified substandard supervisory actions such as controlling workers, reprimanding workers in front of patients and neglect of HWs by the management as influencing their relationships and as highly demotivating [55,57,66]. Poor supervisory practices were reported to affect quality of care as demotivated HWs provided inefficient services [67].

Distrustful, and demotivating, relationships with supervisors and managers were also a result of disrespect, lack of fairness and lack of promotion [49,65,68]. For example, trust was undermined and workers demotivated where managers practised favouritism, bias and discrimination during promotion and allocation of seminar and training opportunities [65] or were perceived not to be transparent in communication [48,51,57,69,70]. “*We are voiceless in this system*” [69] (p. 8) is a quote that exemplifies HW concerns over this sort of disconnection with supervisors. Workers also considered lack of feedback on their performance to be demotivating because they could not know areas that needed improvement [64,66] or because they felt unimportant and undervalued at their workplace—for example due to limited supervision and the lack of supervision criteria [69]. When managers had limited time and interest in HWs’ motivation, trust relationships were limited as workers performed their duties in order to please the managers in exchange for rewards and promotion that would, in turn, act as motivators [49].

### Trust relationships with employing organisation

The term ‘employing organisation’ is used here to refer to the organisation that engages the services of health workers such as the government or body responsible for organisational leadership and human resource management practices. Altogether, 14 articles suggested an association between trust in employing organisation and motivation: 5 reported the positive influence of this trust relationship on motivation [47,48,51,53,57] and 9 reported distrust in the employing organisation as demotivating [50,55,59,60,64,65,67,70,71].

Trust in the employing organisation was evident where transparency and prospects for in-service training motivated HWs to choose working in the public sector over the private sector [47,57]. This had implications for retention. The value of workplace safety for trust and motivation were a major finding in Papua New Guinea where HWs believed that provision of security within health facilities boosted their confidence and enhanced their trust in the government [48]. Being given autonomy and involvement in decision making in the health system also engendered trust relationships between HWs and the employing organisation and was thus considered a motivating factor [47,51,53].

In contrast, lack of support and opportunities for self-empowerment caused strained relationships with employers and demotivated HWs [60]. Reported findings indicated that younger workers were demotivated by their distrust of the health system and management due to inadequate appraisal processes, bureaucratic procedures in promotion and lack of care for their long-term needs [59]. In-depth interviews with clinical officers in Kenya explicitly revealed that the breakdown of trust between them and the central bureaucracy was caused by cases of bribery for promotion and an administration that functioned along ethnic lines during selection for in-service training [59]. Similarly, performance of HWs was negatively affected by lack of trust about government policies and favouritism in selection for in-service training in Malawi [64] and Tanzania [65]. In Ghana, HWs were concerned about unresolved frustrations with the health system that undermined the trust relationships and led to poor quality of care [67].

Poor work conditions, drug shortages and lack of work equipment demotivated HWs because they contributed to poor performance in work-related tasks and thus affected patient care [55,65,70,71]. Poor communication and lack of feedback on policies and guidelines also diminished workplace trust relationships with employers and had negative impact on motivation and performance [50,70].

### Trust relationships with patients

HWs directly highlighted the positive influence of trusting relationships with the patients at the health facilities on their motivation for performance in seven studies [46,49,50,53,63,71,72]. Gaining trust from patients at health facilities was highly ranked as a source of motivation for HWs in an article reporting findings from cross-sectional surveys of public and private sector doctors and nurses in two Indian states [46]. This was the same case in a multi-country study (Burkina Faso, Ghana and Tanzania) that reported workplace trust as developing over time and that it was important in collaboration between HWs and patients [49]. In addition, a study in North Viet Nam identified appreciation and recognition as the most highly ranked motivators [50]. It exemplified appreciation, recognition and respect by patients as words that can be linked to trusting relationships: “*I like my job and I am happy people believe in me. The village HWs trust me, and ask me to help them when needed. I am very proud of that. They are willing to work so it makes me happy. I have retraining and awards every year and the community believes in me. They respect me a lot, so I think I need to work hard for them*” (p. 6).

Conversely, seven studies reported on the negative influence poor trust relationships had on motivation [48,49,57,64,67,71,72]. In a Ghanaian survey, it was reported that HWs displayed their frustration through rudeness, anger, unfriendly behaviour and resentment to patients at health facilities [67]. Poor communication and a language barrier forestalled trusting relationships within health facilities [48,49]. It was reported that lack of trust and respect led to poor communication and was associated with demotivation due to poor interpersonal relations between HWs and patients, especially within rural health facilities [48]. This was linked to lack of cooperation from patients who made guideline and policy implementation difficult for HWs. Studies in Benin, Kenya and Tanzania revealed that lack of trust in patients due to perceived risk of contracting HIV/AIDS and tuberculosis infections led to poor relations with patients and was considered as a demotivating factor [57,71]. Additionally, dissatisfaction with colleagues was reported as a cause of demotivation due to loss of trust from patients resulting from betrayal by colleagues, given by an illustration from a Tanzanian female health worker: “*As a health worker I felt very bad, because we are now ruining our good reputation and losing trust and respect from our patients. Many people who come for HIV test are not comfortable because of not being certain with the issue of confidentiality, and some of them would rather travel to test in another district*” [71] (p. 5). This exemplified the importance of the interaction between workplace trust in colleagues and workplace provider–patient relationship in motivation.

The availability of organisational resources was found to be critical in provider–patient trust relationships and motivation. Staff shortages, heavy workload and resource unavailability were reported to influence the trust relationship due to complaints from patients within the health facilities, and this was reported to affect the quality of care provided [64,71]. HWs indicated that patients used abusive language whenever there were shortages thinking that workers were unwilling to help them, an indication of distrust. Resource constraints and shortages also led to patients’ loss of confidence in HW capacity to provide quality care, further undermining the existing provider–patient relationship [72]. It is important to note that most of the factors that influence provider–patient relationships are bidirectional and therefore it is difficult to delink these two types of trust relationships.

## Discussion

To the authors’ knowledge, this is the first systematic review to gather and analyse evidence on workplace trust relationships and health worker motivation. The conceptual frameworks used in data extraction, categorisation and description of the identified workplace trust relationships allowed for both an open and in-depth approach to this review. Judgements about the suitability of the selected studies may be subject to selection bias, but these judgements were cross-checked between the two authors and the use of the CASP appraisal tool, providing clear guideline on appraisal of selected studies, also limited such bias. The inclusion only of publications available in English may have left out relevant studies published in other languages. Future reviews should consider studies published in other languages to provide relevant evidence from other settings.

This review revealed that workplace trust relationships influence the intrinsic motivation of HWs. Workplace trust had both positive and negative influences over motivation and were reflected in other motivational determinants like recognition, appreciation and rewards; supervision; teamwork; management and welfare support; communication, feedback and openness; and staff shortages, heavy workload and resource unavailability. The review also revealed that interpersonal and organisational factors influence the development of workplace trust relationships. It illuminated the complex nature of these relationships and the manner in which they influence motivation, confirming that motivation is not just a function of a single determinant but rather an output of interactions among various factors [51].

Importantly, no hierarchy was identified among the relationships in terms of the degree of their influence on HW motivation. Instead, these trust relationships appear to interact to influence HW motivation. For example, drug and staff shortages cause tension between HWs and patients leading to distrust of the employing organisation and demotivation [61,71]. Therefore, strategies to enhance the intrinsic motivation of HWs should encompass factors relating to all four of the identified workplace trust relationships.

Some of the articles reviewed provide evidence to suggest that workplace trust also has consequences for intention to leave and quality of care. Intrinsic values and trust relationships between colleagues were reiterated as important predictors of intention to leave [47,48,52,57]. Although there were no clarifications on how trust relates to intention to leave, it is plausible based on other empirical evidence on retention and migration of HWs [7,8].

The review also revealed that positive HW performance and motivation to provide good quality care can be improved by workplace trust relationships that are supportive and respectful [48,50,53,57]. However, poor interpersonal workplace relationships and distrust have the opposite effect on quality of care and performance [58,71]. For example, one study explicitly reported lack of respect between cadres as a cause of distrust, demotivation and the provision of poorer care [58]. Moreover, shortages of drugs and work resources led to low motivation, distrust in the health systems and poor performance by the HWs [71].

There are implications of this review for managerial action. To improve HW performance and quality of care, motivating workplace trust relationships between colleagues can be strengthened through good relationships between cadres, collegial recognition, supportive teamwork, respect and good communication in the workplace [47,51,52,58]. Supervisors and managers also have a major role to play in building workplace trust relationships that promote intrinsic motivation. HWs particularly commended supervisory practices such as supervisor support, recognition and appreciation, fairness in performance, communication and feedback [45,50-53,59,62,66]. These point to the value that sound HRM practices have in establishing and enhancing workplace trust relationships to motivate workers [4].

The employing organisation’s influence over HW motivation cannot be underestimated [27,31]. Its support by provision of work resources—such as drugs, equipment, job safety and security, good working environment and structures, clear job description, and in-service training—allows for the development of trusting behaviour that is critical for performance [50,59]. Research has also demonstrated the relevance of workplace trust to patient experiences [16]. This review supports this relevance by identifying some of the factors of trust that motivate workers to willingly perform their duties and strengthen the bidirectional provider–patient trust relationship. These factors include greater responsibility, respect and appreciation from patients [45,48,50].

Articles reviewed from HICs tended to report positive experiences [43-45,47,52,53] while, in contrast, both positive and negative experiences were identified in LMICs. For LMICs, these findings seem to reflect the wider health sector challenges of resource constraints, inadequate management practices and skills inadequacy [2,4,5,73]. Low remuneration and resource inadequacy were, thus, important influences over workplace distrust and HW demotivation in LMICs along with lack of teamwork, disrespect, lack of support and poor relationships with colleagues, supervisors, managers and patients. Nonetheless, the review also noted that the positive implications of good workplace trust relationships, founded on similar factors, for performance and quality of care are observable in both HICs [44] and LMICs [61].

Theoretical arguments identify trust relationships as critical in the generation and delivery of health care services that establish a wider social value [27,29]. However, low levels of motivation which manifest in ineffective health care delivery can only compound existing health system challenges and weaknesses [4,7,8]. Trust relationships in the health sector, therefore, may act as intrinsic motivators, but lack of trust may lead to disinterest in work itself, which ultimately affects performance [49,52]. When implementing external interventions to motivate HWs, it is necessary to consider the dynamics and nature of workplace trust relationships to avoid undermining existing intrinsic motivation, which is important to performance and may be less expensive to promote than other forms of performance management [4,45].

## Conclusion

The findings in this systematic review highlight the value of workplace trust relationships in influencing the intrinsic motivation of HWs, which is itself a critical and positive influence over HWs’ performances. The review is, therefore, important in contributing to the literature on motivation in the health sector, identifying opportunities for further empirical research and informing policy discussions about how to influence HW motivation to support retention and good quality of health care services.

The review suggests that health systems in different contexts can strengthen workplace trust relationships and intrinsic motivation through positive social interactions, effective communication and good supervisory mechanisms. Professional development activities, training of health workers and organisational and human management practices, processes, resources, structures and culture play critical roles in establishing the positive workplace trust relationships that promote intrinsic motivation.

Yet the evidence in this review also shows that there is limited empirical research on trust and motivation in the health sector. The review is inconclusive on the complex interaction between trust relationships and health worker motivation and their impact on retention, performance and delivery of quality patient care. Therefore, the reviewers recommend further empirical research to investigate this neglected but important aspect of health system strengthening. Further work should also focus on understanding the factors that undermine or strengthen intrinsic motivation in relation to the existing interventions targeting extrinsic motivation, and the broader determinants of motivation.

## Additional files

Additional file 1: **Search strategy.** The search algorithm used in the databases selected for this review. It contains the dates of searches, the search terms, search limits and the search output.
Additional file 2: **Data extraction form.** The form used as data registry and guide for identification and classification of the determinants of health worker motivation.

## Abbreviations

HICs: High-income countries
LMICs: Low- and middle-income countries
HWs: Health workers
HRM: Human resource management

## References

[1] Fritzen S (2007). Strategic management of the health workforce in developing countries: what have we learned?. Hum Resour Health.

[2] Hongoro C, McPake B (2004). How to bridge the gap in human resources for health. Lancet.

[3] Hagopian A, Zuyderduin A, Kyobutungi N, Yumkella F (2009). Job satisfaction and morale in the Ugandan health workforce. Health Aff.

[4] Dieleman M, Gerretsen B, Van Der Wilt GJ (2009). Human resource management interventions to improve health workers’ performance in low and middle income countries: a realist review. Health Res Policy Sys.

[5] Willis-Shattuck M, Bidwell P, Thomas S, Wyness L, Blaauw D, Ditlopo P (2008). Motivation and retention of health workers in developing countries: a systematic review. BMC Health Serv Res.

[6] Bach S (2004). Migration patterns of physicians and nurses: still the same story?. Bull World Health Organ.

[7] Connell J, Zurn P, Stilwell B, Awases M, Braichet J (2007). Sub-Saharan Africa: beyond the health worker migration crisis?. Soc Sci Med.

[8] Blaauw D, Ditlopo P, Maseko F, Chirwa M, Mwisongo A, Bidwell P (2013). Comparing the job satisfaction and intention to leave of different categories of health workers in Tanzania, Malawi, and South Africa. Global Health Act.

[9] Vroom VH (1964). Work and Motivation.

[10] Franco LM, Bennett S, Kanfer R (2002). Health sector reform and public sector health worker motivation: a conceptual framework. Soc Sci Med.

[11] Maslow AH (1943). A theory of human motivation. Psychol Rev.

[12] Herzberg F, Mausner B, Snyderman B (1959). The Motivation to Work.

[13] Kanfer R (1999). Measuring Health Worker Motivation in Developing Countries: Partnerships for Health Reform.

[14] Ryan RM, Deci EL (2000). Intrinsic and extrinsic motivations: classic definitions and new directions. Contemp Educ Psychol.

[15] Dolea C, Adams O (2005). Motivation of health care workers-review of theories and empirical evidence. Cah Sociol Demogr Med.

[16] Gilson L, Palmer N, Schneider H (2005). Trust and health worker performance: exploring a conceptual framework using South African evidence. Soc Sci Med.

[17] Henderson L, Tulloch J (2008). Incentives for retaining and motivating health workers in Pacific and Asian countries. Hum Resour Health.

[18] Eisenberger R, Rhoades L, Cameron J (1999). Does pay for performance increase or decrease perceived self-determination and intrinsic motivation?. J Pers Soc Psychol.

[19] Adams O, Hicks V (2000). Pay and non-pay incentives, performance and motivation. Hum Resour Dev J.

[20] Frey BS, Jegen R (2001). Motivation crowding theory. J Econ Surv.

[21] Chen L, Evans T, Anand S, Boufford JI, Brown H, Chowdhury M (2004). Human resources for health: overcoming the crisis. Lancet.

[22] World Health Organization (2007). Everybody’s Business: Strengthening Health Systems to Improve Health Outcomes: WHO’s Framework for Action.

[23] Simonet D (2008). The New Public Management theory and European health‐care reforms. Can Public Adm.

[24] Newman S, Lawler J (2009). Managing health care under New Public Management. A Sisyphean challenge for nursing. J Soc.

[25] Simonet D. The new public management theory in the British health care system: a critical review. Admin Soc. 2013. doi:10.1177/0095399713485001.

[26] Fox A (1974). Beyond Contract: Work, Power and Trust Relations.

[27] Mechanic D (1998). The functions and limitations of trust in the provision of medical care. J Health Polit Policy Law.

[28] Gilson L (2003). Trust and the development of health care as a social institution. Soc Sci Med.

[29] Gilson L (2006). Trust in health care: theoretical perspectives and research needs. J Health Organ Manag.

[30] Petersen LA, Woodard LD, Urech T, Daw C, Sookanan S (2006). Does pay-for-performance improve the quality of health care?. Ann Intern Med.

[31] Kramer RM (1999). Trust and distrust in organizations: emerging perspectives, enduring questions. Annu Rev Psychol.

[32] Albrecht S, Travaglione A (2003). Trust in public-sector senior management. Int J Hum Resour Manag.

[33] Hall MA, Camacho F, Dugan E, Balkrishnan R (2002). Trust in the medical profession: conceptual and measurement issues. Health Serv Res.

[34] Dixon-Woods M, Agarwal S, Jones D, Young B, Sutton A (2005). Synthesising qualitative and quantitative evidence: a review of possible methods. J Health Serv Res Policy.

[35] Dixon-Woods M, Bonas S, Booth A, Jones DR, Miller T, Sutton AJ (2006). How can systematic reviews incorporate qualitative research? A critical perspective. Qual Res.

[36] Mays N, Pope C, Popay J (2005). Systematically reviewing qualitative and quantitative evidence to inform management and policy-making in the health field. J Health Serv Res Policy.

[37] Bearman M, Dawson P (2013). Qualitative synthesis and systematic review in health professions education. Med Educ.

[38] Dixon-Woods M, Shaw RL, Agarwal S, Smith JA (2004). The problem of appraising qualitative research. Qual Saf Health Care.

[39] Chinchilli VM (2007). General principles for systematic reviews and meta-analyses and a critique of a recent systematic review of long-acting β-agonists. J Allergy Clin Immunol.

[40] Public Health Resource Unit. Critical Appraisal Skills Programme (CASP) making sense of evidence: 10 questions to help you make sense of qualitative research. Oxford 2006. http://www.civilservice.gov.uk/wp-content/uploads/2011/09/Qualitative-Appraisal-Tool_tcm6-7385.pdf. Accessed 27 Sept 2013.

[41] Noyes J, Popay J (2007). Directly observed therapy and tuberculosis: how can a systematic review of qualitative research contribute to improving services? A qualitative meta‐synthesis. J Adv Nurs.

[42] Thomas J, Harden A (2008). Methods for the thematic synthesis of qualitative research in systematic reviews. BMC Med Res Methodol.

[43] Kontodimopoulos N, Paleologou V, Niakas D (2009). Identifying important motivational factors for professionals in Greek hospitals. BMC Health Serv Res.

[44] Kudo Y, Kido S, Shahzad MT, Shida K, Satoh T, Aizawa Y (2010). Enhancing work motivation for Japanese female nurses in small to medium-sized private hospitals by analyzing job satisfaction. Tohoku J Exp Med.

[45] Lambrou P, Kontodimopoulos N, Niakas D (2010). Motivation and job satisfaction among medical and nursing staff in a Cyprus public general hospital. Hum Resour Health.

[46] Peters DH, Chakraborty S, Mahapatra P, Steinhardt L (2010). Job satisfaction and motivation of health workers in public and private sectors: cross-sectional analysis from two Indian states. Hum Resour Health.

[47] Kahler L, Kristiansen M, Rudkjobing A, Strandberg-Larsen M (2012). Surgeons’ motivation for choice of workplace. Dan Med J.

[48] Razee H, Whittaker M, Jayasuriya R, Yap L, Brentnall L (2012). Listening to the rural health workers in Papua New Guinea - the social factors that influence their motivation to work. Soc Sci Med.

[49] Prytherch H, Kagoné M, Aninanya GA, Williams JE, Kakoko DC, Leshabari MT, et al. Motivation and incentives of rural maternal and neonatal health care providers: a comparison of qualitative findings from Burkina Faso, Ghana and Tanzania. BMC Health Services Research. 2013;13(1). doi:10.1186/1472-6963-13-149. 10.1186/1472-6963-13-149 PMC3648439 23617375

[50] Dieleman M, Cuong P, Anh L, Martineau T (2003). Identifying factors for job motivation of rural health workers in North Viet Nam. Hum Resour Health.

[51] Franco LM, Bennett S, Kanfer R, Stubblebine P (2004). Determinants and consequences of health worker motivation in hospitals in Jordan and Georgia. Soc Sci Med.

[52] Hegney D, Plank A, Parker V (2006). Extrinsic and intrinsic work values: their impact on job satisfaction in nursing. J Nurs Manag.

[53] Dickin KL, Dollahite JS, Habicht J (2011). Enhancing the intrinsic work motivation of community nutrition educators: how supportive supervision and job design foster autonomy. J Ambul Care Manage.

[54] Siril H, Hirschhorn LR, Hawkins C, Garcia ME, Li MS, Ismail S (2011). Stress, motivation and professional satisfaction among health care workers in HIV/AIDS care and treatment centers in urban Tanzania: a cross-sectional study. East Afr J Public Health.

[55] Prytherch H, Kakoko DCV, Leshabari MT, Sauerborn R, Marx M. Maternal and newborn healthcare providers in rural Tanzania: in-depth interviews exploring influences on motivation, performance and job satisfaction. Rural Remote Health. 2012;12(3). doi:10.1186/1472-6963-13-149. 22934936

[56] Alhassan RK, Spieker N, van Ostenberg P, Ogink A, Nketiah-Amponsah E, de Wit TFR (2013). Association between health worker motivation and healthcare quality efforts in Ghana. Hum Resour Health.

[57] Mathauer I, Imhoff I. Health worker motivation in Africa: the role of non-financial incentives and human resource management tools. Hum Resour Health. 2006;4(24). doi:10.1186/1478-4491-4-24. 10.1186/1478-4491-4-24 PMC1592506 16939644

[58] Chandler CIR, Chonya S, Mtei F, Reyburn H, Whitty CJM (2009). Motivation, money and respect: a mixed-method study of Tanzanian non-physician clinicians. Soc Sci Med.

[59] Mbindyo P, Gilson L, Blaauw D, English M (2009). Contextual influences on health worker motivation in district hospitals in Kenya. Implemen Sci.

[60] Malik AA, Yamamoto SS, Souares A, Malik Z, Sauerborn R. Motivational determinants among physicians in Lahore, Pakistan. BMC Health Serv Res. 2010;10(201). doi:10.1186/1472-6963-10-201. 10.1186/1472-6963-10-201 PMC2910698 20618962

[61] Greenspan JA, McMahon SA, Chebet JJ, Mpunga M, Urassa DP, Winch PJ (2013). Sources of community health worker motivation: a qualitative study in Morogoro Region, Tanzania. Hum Resour Health.

[62] Dieleman M, Toonen J, Touré H, Martineau T (2006). The match between motivation and performance management of health sector workers in Mali. Hum Resour Health.

[63] Kyaddondo D, Whyte SR (2003). Working in a decentralized system: a threat to health workers’ respect and survival in Uganda. Int J Health Plann Manage.

[64] Manafa O, McAuliffe E, Maseko F, Bowie C, MacLachlan M, Normand C (2009). Retention of health workers in Malawi: perspectives of health workers and district management. Hum Resour Health.

[65] Mubyazi GM, Bloch P, Byskov J, Magnussen P, Bygbjerg IC, Hansen KS (2012). Supply-related drivers of staff motivation for providing intermittent preventive treatment of malaria during pregnancy in Tanzania: evidence from two rural districts. Malar J.

[66] Manongi RN, Marchant TC, Bygbjerg IC (2006). Improving motivation among primary health care workers in Tanzania: a health worker perspective. Hum Resour Health.

[67] Agyepong IA, Anafi P, Asiamah E, Ansah EK, Ashon DA, Narh-Dometey C (2004). Health worker [internal customer] satisfaction and motivation in the public sector in Ghana. Int J Health Plann Manage.

[68] Zinnen V, Paul E, Mwisongo A, Nyato D, Robert A (2012). Motivation of human resources for health: a case study at rural district level in Tanzania. Int J Health Plann Manage.

[69] Kok MC, Muula AS (2013). Motivation and job satisfaction of health surveillance assistants in Mwanza, Malawi: an explorative study. Malawi Med J.

[70] Leshabari MT, Muhondwa E, Mwangu M, Mbembati N (2008). Motivation of health care workers in Tanzania: a case study of Muhimbili National Hospital. East Afr J Public Health.

[71] Mbilinyi D, Daniel ML, Lie GT (2011). Health worker motivation in the context of HIV care and treatment challenges in Mbeya Region, Tanzania: a qualitative study. BMC Health Serv Res.

[72] Campbell C, Scott K, Madenhire C, Nyamukapa C, Gregson S (2011). Sources of motivation and frustration among healthcare workers administering antiretroviral treatment for HIV in rural Zimbabwe. AIDS Care Psychol Socio-Med Asp AIDS HIV.

[73] Rowe AK, de Savigny D, Lanata CF, Victora CG (2005). How can we achieve and maintain high-quality performance of health workers in low-resource settings?. Lancet.

[74] Newton JM, Kelly CM, Kremser AK, Jolly B, Billett S (2009). The motivations to nurse: an exploration of factors amongst undergraduate students, registered nurses and nurse managers. J Nurs Manage.

[75] Agyei-Baffour P, Kotha SR, Johnson JC, Gyakobo M, Asabir K, Kwansah J, et al. Willingness to work in rural areas and the role of intrinsic versus extrinsic professional motivations - a survey of medical students in Ghana. BMC Med Educ. 2011;11(56). doi:10.1186/1472-6920-11-56. 10.1186/1472-6920-11-56 PMC3170278 21827698

[76] de Guzman AB, Dangoy RD, David KCV, Dayo KJH, de Claro KA, von Gerri GG (2009). How many sides does a coin have? A phenomenology of Filipino nurses’ motivation and attitudes toward geriatric care. Educ Gerontol.

[77] Gambino KM (2010). Motivation for entry, occupational commitment and intent to remain: a survey regarding Registered Nurse retention. J Adv Nurs.

[78] Helmink JHM, Kremers SPJ, van Boekel LC, van Brussel-Visser FN, de Vries NK (2012). Factors determining the motivation of primary health care professionals to implement and continue the ‘Beweegkuur’ lifestyle intervention programme. J Eval Clin Pract.

[79] Imai H, Matsuishi K, Ito A, Mouri K, Kitamura N, Akimoto K, et al. Factors associated with motivation and hesitation to work among health professionals during a public crisis: a cross sectional study of hospital workers in Japan during the pandemic (H1N1) 2009. BMC Pub Health. 2010;10(672). doi:10.1186/1471-2458-10-672. 10.1186/1471-2458-10-672 PMC3091577 21050482

[80] Kamanzi J, Nkosi ZZ (2011). Motivation levels among nurses working at Butare University Teaching Hospital. Rwanda Afr J Nurs Midwifery.

[81] Leonard KL, Masatu MC (2010). Professionalism and the know-do gap: exploring intrinsic motivation among health workers in Tanzania. Health Econ.

[82] Lopes JE, Delellis NO (2013). Understanding the motivations of the multigenerational physician assistant workforce. J Am Acad Phys Assist.

[83] Minai MS, Almansour YM (2013). Factors influencing job satisfaction and motivation of nurses in the environment dominated by male nurses. Middle East J Sci Res.

[84] Mubyazi GM, Njunwa K (2013). Perceived impact of health sector reform on motivation of health workers and quality of health care in Tanzania: the perspectives of healthcare workers and district council health managers in four districts. Rwanda J Health Sci.

[85] Negussie N (2012). Relationship between rewards and nurses’ work motivation in Addis Ababa hospitals. Ethiopian J Health Sci.

[86] Serneels P, Lindelow M, Montalvo JG, Barr A (2007). For public service or money: understanding geographical imbalances in the health workforce. Health Policy Plan.

