# Supplementary material for: Exploring the influence of trust relationships on motivation in the health sector: a systematic review
Source: Hum Resour Health. 2015 Mar 31;13:16. doi: 10.1186/s12960-015-0007-5 (PMC4384237; doi:10.1186/s12960-015-0007-5)
Supplement: Additional file 1: — Search strategy. The search algorithm used in the databases selected for this review. It contains the dates of searches, the search terms, search limits and the search output. [file 12960_2015_7_MOESM1_ESM.pdf]

Additional File 1: Search Strategy

Databases	Date of Final Search	Results						
		Total Search	Relevant Articles Retrieved	Combined Databases	After Removal of Duplicates	After Title Screening	After Abstract Screening	Eligible Articles for Review
Africa-Wide Information	17/11/2013	1257	117	1420	1140	204	42	30
Cumulative Index of Nursing & Allied Health (CINHAL)	17/11/2013	3840	172					
PsycINFO	17/11/2013	3893	360					
PubMed	18/11/2013	5338	383					
Scopus	18/11/2013	3389	388					
**TOTAL**		17717	1420	1420	1140	204	42	30

PubMed (383)

((((((((((("Motivation"[Mesh]) OR motivation) OR "Job Satisfaction"[Mesh]) OR job satisfaction) OR "Attitude of Health Personnel"[Mesh]) OR Attitude of Health Personnel) OR staff attitude) OR team work in nursing) OR team work) OR staff retention))

AND

(((((((((((((((((("Trust"[Mesh]) OR trust) OR "Physician-Nurse Relations"[Mesh]) OR Physician-Nurse Relations) OR "Nurse-Patient Relations"[Mesh]) OR Nurse-Patient Relations) OR "Professional-Patient Relations"[Mesh]) OR Professional-Patient Relations) OR "Physician-Patient Relations"[Mesh]) OR Physician-Patient Relations) OR "Dentist-Patient Relations"[Mesh]) OR Dentist-Patient Relations) OR "Patient Care Team"[Mesh]) OR Patient Care Team) OR "Health Services Research"[Mesh]) OR Health Services Research) OR "Organizational Culture"[Mesh]) OR Organizational Culture)

Filters: Journal Article; Free full text available; Full text available; Publication date from 2003/01/01 to 2013/12/31; Humans; English; MEDLINE; Nursing journals; Dental journals

Cumulative Index of Nursing and allied Health (CINHAL) via EBSCOhost (172)

(MM "Motivation+") OR motivation OR trust OR (MM "Trust") OR "job satisfaction" OR (MM "Job Satisfaction+") OR relationship* OR (MM "Interpersonal Relations+") OR (MM "Physician-Patient Relations") OR (MM "Interinstitutional Relations") OR (MM "Dentist-Patient Relations") OR (MM "Patient-Family Relations") OR (MM "Professional-Patient Relations+") OR (MM "Interpersonal Relationships (Omaha)") OR (MM "Employer-Employee Relations+") OR (MM "Teamwork") OR (MM "Management Styles") OR (MH "Attitude of Health Personnel") OR (MH "Employee Attitudes") OR "staff attitude"

AND

OR (MH "Health Personnel, Unlicensed") OR (MH "Personnel, Health Facility") OR (MH "Health Personnel as Patients") OR (MH "Rural Health Personnel") OR (MH "Health Personnel, Infected") OR (MH "Alternative Health Personnel") OR (MH "Health Personnel, Minority") OR (MH "Allied Health Personnel") OR (MH "Health Personnel") OR "health personnel" OR "healthcare provider*"OR "health care provider*" OR "health worker*" OR "healthcare worker*" OR (MH "Hospitals, Public") OR (MH "Hospitals, Pediatric") OR (MH "Hospitals, Psychiatric") OR (MH "Organizations, For Profit") OR (MH "Hospitals, Urban") OR (MH "Hospitals, Special") OR (MH "Hospitals, Rural") OR (MH "Hospitals, Community") OR (MH "Hospitals") OR hospital* OR "health organi?ation*" OR (MH "Health Maintenance Organizations") OR (MH "State Allied Health Organizations") OR (MH "Allied Health Organizations") OR (MH "Organizations, For Profit") OR (MH "Mental Health Organizations") OR (MH "Health Facility Administration") OR "healthcare organi?ation*" OR "health institution*" OR "healthcare institution*" OR (MM "Primary Health Care") OR (MM "Health Care Reform+") OR (MM "Health Care Delivery+") OR "primary health care" OR "healthcare sector" OR "health care sector" OR "health sector" OR (MM "Health Care Industry")

Limiters - Linked Full Text; Abstract Available; Published Date: 2003/01/01 - 2013/12/31

Expanders - Apply related words; also search within the full text of the articles

Search modes - Boolean/Phrase

PsycINFO via EBSCOhost (360)

DE "Motivation" OR MM "Educational Incentives" OR MM "Employee Motivation" OR MM "Extrinsic Motivation" OR MM "Intrinsic Motivation" OR MM "Monetary Incentives" OR Motivation OR DE "Trust (Social Behaviour)" OR Trust OR DE "Employee Attitudes" OR DE "Satisfaction" OR DE "Career Change" OR DE "Employee Engagement" OR DE "Employee Retention" OR DE "Job Enrichment" OR DE "Job Involvement" OR DE "Organizational Commitment" OR DE "Quality of Work Life" OR DE "Role Satisfaction" OR “Job Satisfaction” OR DE "Relationship Satisfaction" OR DE "Interpersonal Relationships" OR Relationship OR Teamwork OR Retention

AND

DE "Professional Personnel" OR DE "Allied Health Personnel" OR DE "Medical Personnel" OR “Health Personnel” OR "Healthcare Provider*" OR "Health Care Provider*" OR "Healthcare Worker*" OR "Health Worker*" OR DE "Hospitals" OR Hospitals OR "Health Organi?ation*" OR "Healthcare Organi?ation*" OR "Health Care Organi?ation*" OR "Health Institution*" OR "Healthcare Institution*" OR "Health Care Institution*" OR DE "Primary Health Care" OR DE "Health Care Utilization" OR DE "Health Care Reform" OR DE "Health Care Policy" OR DE "Health Care Delivery" OR DE "Health Care Services" OR DE "Health Care Administration" OR "Primary Health Care" OR "Health Sector" OR "Healthcare Sector" OR "Health Care Sector" OR "Healthcare Industry" OR "Health Care Industry"

Africa-Wide Information via EBSCOhost (117)

KW motivation OR SM motivation OR KW trust OR TX "job satisfaction" OR TX relationship* OR TX teamwork OR TX "staff attitude*" OR TX retention

AND

KW "health personnel" OR TX "healthcare provider*" OR TX "health care provider*" OR KW "health worker*" OR TX "healthcare worker" OR TX "health care worker*" OR SM hospital* OR KW "health organi?ation*" OR TX "health care organi?ation*" OR TX "healthcare organi?ation*" OR TX "healthcare institution*" OR TX "health care institution*" OR TX "health institution*" OR TX "primary health care" OR TX "health care sector" OR TX "healthcare sector" OR KW "health sector" OR TX "healthcare industry"

Limiters - Year Published: 2003-2013; Language: English

Search modes - Boolean/Phrase

Scopus (388)

((TITLE-ABS-KEY(motivation)) OR (TITLE-ABS-KEY(trust)) OR (ALL("job satisfaction")) OR (ALL(teamwork)) OR (ALL("staff attitude")) OR (ALL("provider-patient relationship")) OR (ALL("patient-provider relationship")) (ALL(retention)) OR (TITLE-ABS-KEY(retention)))

AND

(((ALL("health personnel")) OR (TITLE-ABS-KEY("health personnel")) OR (ALL("healthcare providers")) OR (ALL("health care providers")) OR (ALL("healthcare workers")) OR (ALL("health care workers")) OR (ALL("health workers"))) OR ((TITLE-ABS-KEY(hospital)) OR (ALL("health organi?ation")) OR (ALL("health care organi?ation")) OR (ALL("healthcare organi?ation")) OR (ALL("healthcare institution")) OR (ALL("health care institution")) OR (ALL("health institution"))) OR ((ALL("primary health care")) OR (ALL("health care sector")) OR (ALL("healthcare sector")) OR (ALL("health sector")) OR (ALL("healthcare industry"))))

AND

(LIMIT-TO(PUBYEAR, 2013) OR LIMIT-TO(PUBYEAR, 2012) OR LIMIT-TO(PUBYEAR, 2011) OR LIMIT-TO(PUBYEAR, 2010) OR LIMIT-TO(PUBYEAR, 2009) OR LIMIT-TO(PUBYEAR, 2008) OR LIMIT-TO(PUBYEAR, 2007) OR LIMIT-TO(PUBYEAR, 2006) OR LIMIT-TO(PUBYEAR, 2005) OR LIMIT-TO(PUBYEAR, 2004) OR LIMIT-TO(PUBYEAR, 2003)) AND (LIMIT-TO(DOCTYPE, "ar") OR LIMIT-TO(DOCTYPE, "re") OR LIMIT-TO(DOCTYPE, "ip") OR LIMIT-TO(DOCTYPE, "ar") OR LIMIT-TO(DOCTYPE, "re") OR LIMIT-TO(DOCTYPE, "ip")) AND (LIMIT-TO(LANGUAGE, "English")) AND (LIMIT-TO(SRCTYPE, "j"))
