# Supplementary material for: Exploring the influence of trust relationships on motivation in the health sector: a systematic review
Source: Hum Resour Health. 2015 Mar 31;13:16. doi: 10.1186/s12960-015-0007-5 (PMC4384237; doi:10.1186/s12960-015-0007-5)
Supplement: Additional file 2: — Data extraction form. The form used as data registry and guide for identification and classification of the determinants of health worker motivation. [file 12960_2015_7_MOESM2_ESM.pdf]

Additional file 2: Data Extraction Form

**Author(s) & Year of Publication**	**Internal/Individual Determinants**		**Organisational/Institutional Determinants**		**Socio-Cultural Determinants**		**Relationship Types Identified**	**Trust Issues Identified**
	**Motivators**	**Demotivators**	**Motivators**	**Demotivators**	**Motivators**	**Demotivators**		
Agyepong et al 2003 [1]			Additional duty hours allowance; In-service training	Low salaries; Lack of essential equipment, tools and supplies for work; Lack of work transport; Delayed promotions; Inadequate staffing; Housing; Inequitable additional duty hours allowance		Effect of the job on family; Social isolation and loneliness		Outward display of frustration through rudeness, anger, unfriendly behavior and resentment to patients
Alhassan et al 2013[2]			Availability of drugs and medical resources;	Low salaries; Lack of transport to work; Inadequate resources; Lack of career development prospects			Poor relations due to resource inadequacy	Trusting relations through professionalism
Campbell et al 2011[3]	Altruism; Achievement/patient recoveries; Personal experiences with HIV; Pride			Staff shortages; Inadequate drugs and supplies;			Relationship with patients	Losing confidence to patients
Chandler et al 2009[4]	Altruism; Pride; Respect			Low salaries; Poor working conditions;	Social status expectations			Patient respect and appreciation; Lack of respect between ranks

Dickin, Dollahite & Habicht 2011[5]	Altruism/desire to help; Autonomy/freedom; Pride		Supportive supervision; Benefits	Work overload; Low pay;			Caring relationships with participants; Relationships with supervisors; Teamwork and good relationships with colleagues	Trust with supervisor; Respect from supervisor
Dieleman et al 2003 [6]	Respect; Job and income stability; love for work		Recognition; Appreciation and support by managers and colleagues; In-service training	Low income and allowance; Difficult transportation; Lack of updated information and knowledge; Heavy workload; Supervision without feedback; Inadequate performance appraisal	Community appreciation and respect		Recognition from colleagues, managers, and clients; Good relationships with managers and colleagues at workplace. Relationships with community	People believe in HWs, people ask for help when needed and respect them; I need to work hard for them; Inviting health workers to participate in community activities and parting in health educational campaigns
Dieleman et al 2006[7]	Feeling responsible;	Partner living far away; Living far away from urban centre and decision making places	In-service training: Recognition and appreciation	Lack of material and equipment; Lack of recognition; Difficult living conditions; Lack of job descriptions; Subjective: Poor functioning of the health committee performance appraisal; Poor management; Lack of rewards			Recognition and appreciation by superiors, colleagues and patients	Having good colleagues; Holding staff responsible

Franco et al 2004[8]	Self-efficacy/desire for achievement; Work locus of control (goals and preferences); Pride; Attitudes to change (motives); Autonomy: Values (work, self-respect)		Organizational citizenship behavior; Management openness; Resource availability; Job properties; Regular feedback; Organizational culture; Management practices; Communication ; Recognition; Financial rewards	Lack of transparency in performance appraisals; Little opportunity for promotion;	Societal values and beliefs; Culture and organizational function; Culture and provider-patient R/ship; Social respect		Teamwork; social respect; increasing pride through recognition by hospital and community; provider-patient relationship; work relationships within the organization	Communication; Management support; Recognition; Organizational citizenship through rewards to encourage teamwork; Managers perceived to be less open
Greenspan et al 2013[9]	Altruism/ dedication to public service; Knowledge gained		Financial incentives; Work equipment and supplies; In-service training; Adequate supervision	Inadequate remuneration; Inadequate supplies		Moral, monetary, material and work-related support from family; Recognition from society	Relationships with community; Relationship with supervisor	Insults from community members; Trusting supervision
Hegney, Plank & Parker 2006[10]		Emotional challenges; Lack of autonomy; High work stress; Physical demands of work	Teamwork and support; Availability of equipment for work; Career advancement prospects	Poor remuneration; Lack of rewards for skills and experience; Inconvenient ; working hours; Workplace safety		Poor regard of nursing by community	Teamwork and support from colleagues	
Kahler et al 2012[11]	Influence on the job/autonomy; Patient-centred care		Transparency				Good relationships with colleagues	

Kok & Muula 2013[12]				Low salary; Poor access to training; Heavy workload; Extensive job description; Lack of supervision, communication & transport.		Low recognition	Relationship with supervisors and management	
Kontodimopoulos et al 2009[13]	Achievements (pride, appreciation, respect)		Remuneration; Relationship with co-workers			Social acceptance	Relationships with supervisors and colleagues; Professional relationships	Appreciation, respect and social acceptance
Kudo et al 2010[14]	Pride; Altruism; Personal growth			Poor communication; Low salaries; Workplace safety; Poor working relationships with superiors and colleagues			Relationship with superiors and colleagues	
Kyaddondo & Whyte 2003[15]	Ability to help family and relatives		Lack of professional autonomy	Unclear employment terms and conditions of service; Lack of employment confirmation; Low remuneration; Lack of promotions; Resource constraints; Lack of in-service training opportunities; Shortage of equipment and supplies		Lack of respect due to supervision by community members;	Diminished social value and respect due to supervision by community and loss of professional autonomy (provider-patient relationship)	Suspicion and circumscribing freedom of action by community due to closer community monitoring; Use of professional skills for respect and mutual social relations

Lambrou et al 2010[16]	Respect; Job meaningfulness ; Pride		Relationships with supervisors and colleagues; Salary; Work Environment		Social acceptance; Appreciation		Interpersonal relationships with supervisors and colleagues	Respect; Pride
Leshabari et al 2008[17]				Low salaries; Working environment; Inadequate equipment and facilities; Lack of job description; Lack of feedback; Infrequent job performance evaluation; Lack of rewards; No in-service training; Poor intra-organizational communication; Lack of inclusion in decision making			Lack of concern by employers for staff welfare	Lack of intra-organizational communication
Malik et al 2010[18]	Altruism/serving people; Work interest; Career growth; Ability to support oneself and family; Autonomy; Empowerment	Less career growth; Inability to support oneself and family	Opportunities for higher qualification; Good working environment; Personal safety; Good pay and other financial incentives	Poor supervision; Few opportunities for career development; Resource unavailability; Poor working conditions; Less pay; Heavy workload and long working hours	Social rewards; Respect	Disrespect; Poor interpersonal relations; Less social rewards; Less social and personal time	Disrespect, poor Interpersonal relations	
Manafa et al 2009[19]	Low cost of living in rural area;			Lack of job descriptions; Unfair selection for continuous education and in-service training; Lack of rewards; High workloads; Lack of supplies and			Relationship with patients	

				equipment; Inadequate supervision; Lack of autonomy; Lack of performance appraisal; Limited opportunities for career progression				
Manongi, Merchant & Bygbjerg 2006[20]				Understaffing; Unclear job description (acting upwards or downwards);Gambling with health due to inadequate work facilities; Lack of in-service training; Lack of feedback after referrals; Inadequate supervision; Lack of promotions			Lack of inter-professional exchange (during referrals); Lack of professional identity and recognition by employer and community	Distrust regarding promotion; To be trusted by community important for motivation
Mathauer & Imhoff 2006[21]	Professional and vocational conscience;Altruism;		Recognition by supervisors; Communication and feedback; Good work environment; Means and material availability; In-service training; Regular remuneration; Appreciation and rewards		Recognition by community		Recognition by community; Unhelpful and distant supervision rather than personal and supportive; Inadequate communication and bad treatment of staff	Reserved reaction towards patients with HIV because of fear of infection; Appreciation by patients; Envy among colleagues

Mbilinyi, Daniel & Lie 2011[22]	Altruism/desire to help; Responsibility	Perceived risk of contracting HIV and tuberculosis	Essential drugs; Sufficient infrastructure; Human resources	Lack of acknowledgement and appreciation; Lack of clear job description; Shortage of essential supplies; Overtime work without pay of non-financial incentives; Staff shortages; Lack of fairness	Support & cooperation from community	Undervalued by community	Relationship with patient (perceived stigma)	Patients blame HWs for poor services; Lack of trust between colleagues
Mbindyo et al 2009[23]	Altruism; prestige; Job security	Unmet expectations	Supportive managers	Staff shortages; Non-medical supplies and drug shortages; Relationships with colleagues; Lack of fairness in equal access to training opportunities; Lack of incentives; Lack of recognition and appreciation; Inadequate communication; Lack of promotions; Low salaries; Poor schemes of service; Lack of career development		Negative attitude from the community	Relationships with colleagues and supervisors; Relationship with patients; Poor teamwork across cadres	Young workers not trusting the system; Breakdown of trust between workers and central bureaucracy
Mubyazi et al 2012[24]				Understaffing; Increased workload; Less supportive supervision; Limited career development opportunities; Poor health facility infrastructure			Relationship with supervisor	

Newton et al 2009[25]	Desire to help; Caring motive; Sense of achievement (positive acknowledgement)				Recognition and appreciation by patients		Relationship with patients	
Peters et al 2010[26]			Job location; Income levels; Training opportunities; Tools for job; Good employment benefits; Recognition; Good supervision	Work demands;	Time for family life		Relationships with colleagues	Trusted by clients
Prytherch et al 2012[27]			In-service training;	Understaffing; Heavy workload; Lack of promotion; Work environment; Low pay; Poor security; Lack of job description; Poor supervision and performance appraisal; Lack of autonomy; Unfair promotions	Community appreciation	Lack of professional association	Relationship with community (congratulate & appreciate HW); Relationship with supervisor; Relationship with patients	Blames by supervisor; Mistrust; Patients blames management
Prytherch et al 2013[28]	Altruism; Appreciation		Good supervision; Support from managers; Transparent salary levels; Salary increase & retirement benefits	Inadequate living conditions; Lack of incentives; Lack of job description; Heavy workload; Difficult working conditions			Relationships with managers, supervisors, colleagues and patients	Asking colleague for help; Keeping quiet when unsure

Razee et al 2012[29]		Meeting family obligations; Inadequate Personal safety and security		Workplace safety	Support from local community; Respect and appreciation by patients and community; Social belonging	Societal expectation on gender roles	Relationships with colleagues and patients	Lack of trust and respect; Trust for community cooperation
Siril et al 2011[30]	Altruism/desire to help	Emotional drainage	Adequate supervision; Feedback and performance appraisal; In-service training; Availability of resources; Teamwork; Good working environment; Adequate support; Availability of information				Teamwork; Good relationship with supervisors	Understanding each other; Respect
Zinnen et al 2012[31]	Job security		Career development; Supportive supervision	Inadequate salary & retirement benefits; Inadequate basic working equipment; Staff shortages; Work overload; Lack of services; Favouritism; Inadequate promotions			Relationship with management Relationship with supervisor	Disrespect, harsh language

References

1. Agyepong IA, Anafi P, Asiamah E, Ansah EK, Ashon DA, Narh-Dometey C: **Health worker [internal customer] satisfaction and motivation in the public sector in Ghana.** Int J Health Plann Manage 2004, **19**(4):319-336.

2. Alhassan RK, Spieker N, van Ostenberg P, Ogink A, Nketiah-Amponsah E, de Wit TFR: **Association between health worker motivation and healthcare quality efforts in Ghana.** Hum Resour Health 2013, **11**(1).

3. Campbell C, Scott K, Madenhire C, Nyamukapa C, Gregson S: **Sources of motivation and frustration among healthcare workers administering antiretroviral treatment for HIV in rural Zimbabwe.** AIDS Care Psychol Socio-Med Asp AIDS HIV 2011, **23**(7):797-802.

4. Chandler CIR, Chonya S, Mtei F, Reyburn H, Whitty CJM: **Motivation, money and respect: a mixed-method study of Tanzanian non-physician clinicians.** Soc Sci Med 2009, .

5. Dickin KL, Dollahite JS, Habicht J-: **Enhancing the intrinsic work motivation of community nutrition educators: How supportive supervision and job design foster autonomy.** J Ambul Care Manage 2011, **34**(3):260-273.

6. Dieleman M, Cuong P, Anh L, Martineau T: **Identifying factors for job motivation of rural health workers in North Viet Nam.** Human Resources for Health 2003, **1**.

7. Dieleman M, Toonen J, Touré H, Martineau T: **The match between motivation and performance management of health sector workers in Mali.** Hum Resour Health 2006, **4**(2):00035-00033.

8. Franco LM, Bennett S, Kanfer R, Stubblebine P: **Determinants and consequences of health worker motivation in hospitals in Jordan and Georgia.** Soc Sci Med 2004, **58**(2):343-355.

9. Greenspan JA, McMahon SA, Chebet JJ, Mpunga M, Urassa DP, Winch PJ: **Sources of community health worker motivation: A qualitative study in Morogoro Region, Tanzania.** Hum Resour Health 2013, **11**(1).

10. Hegney D, Plank A, Parker V: **Extrinsic and intrinsic work values: Their impact on job satisfaction in nursing.** J Nurs Manage 2006, **14**(4):271-281.

11. Kahler L, Kristiansen M, Rudkjobing A, Strandberg-Larsen M: **Surgeons' motivation for choice of workplace.** Dan Med J 2012, **59**(9):A4508.

12. Kok MC, Muula AS: **Motivation and job satisfaction of health surveillance assistants in Mwanza, Malawi: An explorative study.** Malawi Med J 2013, **25**(1):5-11.

13. Kontodimopoulos N, Paleologou V, Niakas D: **Identifying important motivational factors for professionals in Greek hospitals.** BMC health services research 2009, **9**(1):164.

14. Kudo Y, Kido S, Shahzad MT, Shida K, Satoh T, Aizawa Y: **Enhancing work motivation for Japanese female nurses in small to medium-sized private hospitals by analyzing job satisfaction.** Tohoku J Exp Med 2010, **220**(3):237-245.

15. Kyaddondo D, Whyte SR: **Working in a decentralized system: a threat to health workers' respect and survival in Uganda.** Int J Health Plann Manage 2003, **18**(4):329-342.

16. Lambrou P, Kontodimopoulos N, Niakas D: **Motivation and job satisfaction among medical and nursing staff in a Cyprus public general hospital.** 2010, .

17. Leshabari MT, Muhondwa EPY, Mwangu MA, Mbembati NAA, M TL, Muhondwa E, M AM, Mbembati N: **Motivation of health care workers in Tanzania: a case study of Muhimbili National Hospital.** *East African Journal of Public Health* 2008, **5; 5**(1; 1)**:**32; 32-37; 37.

18. Malik AA, Yamamoto SS, Souares A, Malik Z, Sauerborn R: **Motivational determinants among physicians in Lahore, Pakistan.** BMC Health Serv Res 2010, **10**:201-6963-10-201.

19. Manafa O, McAuliffe E, Maseko F, Bowie C, MacLachlan M, Normand C: **Retention of health workers in Malawi: perspectives of health workers and district management.** Human Resources for Health 2009, **7**(1):65.

20. Manongi RN, Marchant TC, Bygbjerg IC: **Improving motivation among primary health care workers in Tanzania: a health worker perspective.** Human resources for health 2006, **4**(1):6.

21. Mathauer I, Imhoff I: **Health worker motivation in Africa: the role of non-financial incentives and human resource management tools.** Human resources for health 2006, **4**(1):24.

22. Mbilinyi D, Daniel ML, Lie GT: **Health worker motivation in the context of HIV care and treatment challenges in Mbeya Region, Tanzania: a qualitative study.** BMC Health Serv Res 2011, **11**:266-6963-11-266.

23. Mbindyo P, Gilson L, Blaauw D, English M: **Contextual influences on health worker motivation in district hospitals in Kenya.** Implementation Science 2009, **4**(1):43.

24. Mubyazi GM, Njunwa K: **Perceived Impact of Health Sector Reform on Motivation of Health Workers and Quality of Health Care in Tanzania: the Perspectives of Healthcare Workers and District Council Health Managers in Four Districts.** Rwanda Journal of Health Sciences 2013, **2**(1):43-54.

25. Newton JM, Kelly CM, Kremser AK, Jolly B, Billett S: **The motivations to nurse: An exploration of factors amongst undergraduate students, registered nurses and nurse managers.** J Nurs Manage 2009, **17**(3):392-400.

26. Peters DH, Chakraborty S, Mahapatra P, Steinhardt L: **Job satisfaction and motivation of health workers in public and private sectors: Cross-sectional analysis from two Indian states.** Hum Resour Health 2010, **8**.

27. Prytherch H, Kakoko DC, Leshabari MT, Sauerborn R, Marx M: **Maternal and newborn healthcare providers in rural Tanzania: in-depth interviews exploring influences on motivation, performance and job satisfaction.** Rural Remote Health 2012, **12**:2072.

28. Prytherch H, Kagone M, Aninanya GA, Williams JE, Kakoko DC, Leshabari MT, Ye M, Marx M, Sauerborn R: **Motivation and incentives of rural maternal and neonatal health care providers: a comparison of qualitative findings from Burkina Faso, Ghana and Tanzania.** BMC Health Serv Res 2013, **13**:149-6963-13-149.

29. Razee H, Whittaker M, Jayasuriya R, Yap L, Brentnall L: **Listening to the rural health workers in Papua New Guinea - The social factors that influence their motivation to work.** Soc Sci Med 2012, **75**(5):828-835.

30. Siril H, Hirschhorn LR, Hawkins C, Garcia ME, Li MS, Ismail S, Mdingi SG, Chalamilla G, Fawzi W, Kaaya S: **Stress, motivation and professional satisfaction among health care workers in HIV/AIDS care and treatment centers in urban Tanzania: a cross-sectional study.** East Afr J Public Health 2011, **8**(1):17-24.

31. Zinnen V, Paul E, Mwisongo A, Nyato D, Robert A: **Motivation of human resources for health: A case study at rural district level in Tanzania.** Int J Health Plann Manage 2012, **27**(4):327-347.
